# Supplementary material for: Transcultural Validation of a Spanish Version of the Quality of Life in Epidermolysis Bullosa Questionnaire
Source: Int J Environ Res Public Health. 2022 Jun 9;19(12):7059. doi: 10.3390/ijerph19127059 (PMC9222315; doi:10.3390/ijerph19127059)
Supplement: Supplementary file 1 [file ijerph-19-07059-s001.zip › ijerph-1720407-supplementary.pdf]

CUESTIONARIO CALIDAD DE VIDA EN EPIDERMÓLISIS BULLOSA (QOLEB)

Por favor, conteste estas preguntas respecto a la forma en que la Epidermólisis bullosa (EB) afecta a su vida. Escoja marcando con una ☒ la opción que más se parezca a su situación actual. Por favor, anote cuánto tardó en completar este cuestionario al final del mismo.

Muchas gracias por su colaboración.

1. ¿Tu EB afecta a tu capacidad para moverte por casa?
- ☐ Para nada
- ☐ Un poco
- ☐ Bastante
- ☐ Severamente

2. ¿Tu EB afecta a tu capacidad para bañarte o ducharte?
- ☐ No, no me afecta
- ☐ Sí, en ocasiones necesito ayuda
- ☐ Sí, necesito ayuda la mayoría de las veces
- ☐ Sí, necesito apoyo cada vez que me baño-ducho

3. ¿Tu EB te causa dolor físico?
- ☐ No me causa dolor
- ☐ Dolor ocasional
- ☐ Dolor frecuente
- ☐ Dolor constante

4. ¿Cómo afecta tu EB a tu capacidad para escribir?
- ☐ No me afecta para escribir
- ☐ Me resulta difícil sujetar el bolígrafo
- ☐ Me resulta más fácil teclear que escribir
- ☐ No puedo escribir debido a mi EB

5. ¿Tu EB afecta a tu capacidad para comer?
- ☐ No, como con normalidad
- ☐ Un poco
- ☐ Bastante
- ☐ Dependo de mi gastrostomía para mi nutrición

6. ¿Tu EB afecta a tu capacidad para ir de compras?
- ☐ No, en ningún caso
- ☐ Un poco
- ☐ Bastante
- ☐ Siempre necesito ayuda

7. ¿Cómo te afecta tu EB a la hora de realizar deporte?
- ☐ No me afecta
- ☐ Tengo que tener cuidado al hacer deporte
- ☐ Tengo que evitar algunos deportes
- ☐ Tengo que evitar todos los deportes

8. ¿Cómo de frustrado/a te sientes debido a tu EB?
- ☐ Nada frustrado/a
- ☐ Un poco
- ☐ Bastante
- ☐ Tan frustrado/a que estoy enfadado/a la mayor parte del tiempo

9. ¿Tu EB afecta a tu capacidad para moverte fuera de la casa?
- ☐ No, en ningún caso
- ☐ Un poco
- ☐ Bastante
- ☐ Severamente

10. ¿Cómo afecta tu EB la manera en la que te relacionas con los demás miembros de tu familia?
- ☐ No tiene impacto
- ☐ Un impacto leve
- ☐ Un gran impacto
- ☐ Un impacto muy grande

11. ¿Cómo de avergonzado/a te hacen sentir las personas debido a tu EB?
- ☐ Nada avergonzado/a
- ☐ Un poco
- ☐ Mucho
- ☐ Extremadamente

12. ¿Has necesitado, o necesitas adaptar tu casa (instalar rampas, etc.) debido a tu EB?
- ☐ No, en absoluto
- ☐ Alguna adaptación pequeña
- ☐ Muchas adaptaciones
- ☐ Se ha tenido que adaptar la casa completa

13. ¿Afecta tu EB a tu relación con amigos?
- ☐ No, en absoluto
- ☐ Un poco
- ☐ Bastante
- ☐ Restringe severamente mi interacción social

14. ¿Cómo de preocupado/a o angustiado/a te sientes debido a tu EB?
- ☐ En absoluto angustiado/a
- ☐ Un poco
- ☐ Bastante
- ☐ Extremadamente

15. ¿Cómo te afecta la EB, a ti o a tu familia, económicamente?
- ☐ No tiene impacto
- ☐ Afecta ligeramente
- ☐ Afecta bastante
- ☐ Afecta seriamente

16. ¿Cómo de deprimido/a te sientes debido a tu EB?
- ☐ Nada
- ☐ Un poco
- ☐ Bastante
- ☐ Constantemente deprimido/a

17. ¿Cómo de incómodo/a te hacen sentir los demás (por ejemplo, burlas o miradas fijas) debido a tu EB?
- ☐ En absoluto
- ☐ Poco
- ☐ Bastante
- ☐ Tanto que no salgo a relacionarme socialmente

¿Cuánto tardó en completar este cuestionario? .... minutos.
